# Pattern of Surgical Diseases Affecting Females in a Teaching Hospital in Central India: A Demographic Study

**DOI:** 10.1055/s-0043-1770953

**Published:** 2023-07-10

**Authors:** Shehtaj Khan, Vishal Bansal, Sakshi Goyal

**Affiliations:** 1 Department of Emergency Medicine, Peoples Medical College and Research Centre, Bhopal, Madhya Pradesh, India; 2 Department of General Surgery, Nandkumar Singh Chouhan Government Medical College, Khandwa, Madhya Pradesh, India

**Keywords:** female health, gender bias, surgical diseases, cholelithiasis, breast diseases

## Abstract

**Introduction** Despite progress in eliminating the social and health disparity between men and women during the last century, gender equality remains an elusive goal, particularly in the developing world. This gender-based bias has been found to directly result into poor health outcome in females. Hence, it is vital to know the number and pattern of surgical diseases affecting females in any setup, so as to improve their admission rates and reach out to this neglected half of population.

**Materials and Methods** This was a demographic study done at a teaching hospital in Central India from January to June 2020. Data of patients discharged from female surgery ward were collected from medical record department. Age, diagnosis, urban–rural distribution, and length of hospital stay of patients were noted, and data were analyzed statistically.

**Results** A total of 187 patient records were studied, which revealed that the mean age of the patients was 40.35 years; maximum patients were of gastrointestinal surgery (53.42%) in which the most common diagnosis was cholelithiasis (25.13%). Urological diseases (15.50%), breast diseases (12.83%), perianal disease (9.09%), and thyroid diseases (5.34%) were found in decreasing order of frequency. Overall hospital stays of patients ranged from 1 to 14 days with average stay of 6.35 days.

**Conclusion** In our study, cholelithiasis was found to be the most common surgically treated disease followed by urological diseases. Breast symptoms, although commonly affecting females, did not turn into admissions as there remains a social taboo attached to it. Breast cancer still presents late, despite being the most common cancer in females in India. Approximately 65% patients were discharged within first 5 days of their admission, which indicates good hospital care and improves patient satisfaction levels. Still there is greater need for public health efforts to improve the monitoring, safety, and availability of surgical services to female patients.

Health is very important, as it contributes to human well-being and economic process. 1 Various factors like culture, socioeconomic status, and geographical location affect health in India. 2 Gender is an important factor in most of the social determinants of health—which embrace social, economic, and political factors that play a significant role within the health outcomes of female in India and access to health care in India. Studies have observed that males visit hospital more frequently than females, and hospital admission rates have dramatic gender-based variations. 3 This gender-based bias has been found to directly result into poor health outcomes in females. 4

Currently, females in India face a large number of health issues that ultimately have an effect on the country's economic output. It is important to address the disparities that exist in our health care system to improve outcome, as it is only through the creation of quality human resource that we can gain economically.

Surgery has become an integral part of world health care, with calculable estimated 234 million operations performed yearly. According to the World Bank 2002 report, nearly 11% of the diseases burden, calculated in Disability Adjusted Life Years, was that of surgically treatable conditions. 5

It is vital to know the number and pattern of surgical diseases in any setup, so as to improve its safety and patient care. Detailed incidence and prevalence data regarding different diagnosis are extremely important in determining community surgical needs and in assessing the potential impact of intervention strategies. Hence, the aim of this study was to analyze the demographics of female patients suffering from various surgical diseases burden in a teaching hospital in Central India.

## Materials and Methods

It was a demographic study done at a teaching hospital in Central India from January 2020 to June 2020. Data of all discharged patients admitted in female surgery ward were studied. Age, diagnosis, urban–rural distribution, and length of hospital stay were noted.

### Inclusion Criteria

- All female patients admitted in female general surgery ward.

### Exclusion Criteria

- Intensive care unit patients.
- Super specialty surgical patients admitted in separate wards.
- Patients discharged from casualty ward.

Ethical approval was taken from institutional ethical committee.

Statistical analysis plan in which data were tabulated and evaluated in percentage, mean, standard deviation, and range using appropriate statistical tests.

## Results

A total of 187 patient records were studied, and information collected about their age, diagnosis, urban or rural, new or follow-up patients, treatment given, and total length of hospital stay. The mean age of the patients was 40.35 ± 14.22 (standard deviation [SD]) years with age ranging from 18 to 82 years. Maximum patients (25.67%) were in 21 to 30 years age group ( Table 1 ).

**Table 1:** Age distribution of total patients with different diseases

Age group (y)	Cholelithiasis patients *n* (%)	Urological disease patients *n* (%)	Breast disease patients *n* (%)	Perianal disease patients *n* (%)	Thyroid disease patients *n* (%)	Others *n* (%)	Overall patients *n* (%)
<20	0	3 (10.34)	2 (8.33)	1 (5.88)	0	10 (16.39)	16 (8.55)
21–30	4 (8.51)	9 (31.03)	9 (37.5)	4 (23.52)	1 (10)	21 (34.42)	48 (25.67)
31–40	20 (42.55)	8 (27.58)	3 (12.5)	3 (17.64)	4 (40)	8 (13.11)	46 (24.60)
41–50	10 (21.27)	6 (20.68)	7 (29.16)	4 (23.52)	4 (40)	9 (14.75)	40 (21.40)
51–60	8 (17.02)	1 (3.44)	2 (8.33)	4 (23.52)	0	7 (11.47)	22 (11.77)
61–70	4 (8.51)	2 (6.88)	1 (4.16)	0	1 (10)	4 (6.55)	12 (6.42)
71–80	1 (2.12)	0	0	0	0	1 (1.63)	2 (1.06)
81–90	0	0	0	1 (5.88)	0	0	1 (0.53)
Total	47 (100)	28 (100)	24 (100)	17 (100)	10 (100)	61 (100)	187 (100)

Maximum patients were of gastrointestinal surgery (53.42%) in which the most common was cholelithiasis (25.13%), followed by urological diseases (15.50%), breast diseases (12.83%), perianal disease (9.09%), thyroid diseases (5.34%), and others (3.73%) ( Tables 2 , 3 , 4 , 5 , 6 , 7 ).

**Table 2:** Diagnosis, mean age with standard deviation (±SD), mean hospital stay standard deviation (±SD), ratio of locality of patients and management

S. no.	Diagnosis	*n* (%)	Mean age ± SD (range; y)	Mean hospital stay ± SD (range; d)	Urban–rural ratio	Management	
	Gastrointestinal surgery	100 (100%)				Operative	Conservative
1.	Cholelithiasis	47 (47)	44.73 ± 11.59 (26–67)	5 ± 1.73 (3–11)	41:6	40	7
2.	Appendicitis	20 (20)	29.3 ± 12.02 (18–42)	5.2 ± 1.20 (3–8)	3:1	14	6
3.	Subacute intestinal obstruction	7 (7)	49.28 ± 16.08 (29–75)	7.85 ± 2.84 (5–12)	5:2	2	5
4.	Stoma closure	7 (7)	30 ± 8.73 (27–47)	6.85 ± 1.95 (4–10)	2:5	7	0
5.	Ileal perforation	5 (5)	40.6 ± 12.69 (25–58)	7.8 ± 2.31 (4–11)	3:2	5	0
6.	Incisional hernia	5 (5)	46.8 ± 10.51 (33–62)	6 ± 1.41 (4–8)	4:1	5	0
7.	Choledocholithiasis	3 (3)	50.6 ± 10.20 (42–65)	4.33 ± 0.94 (3–5)	2:1	3	0
8.	Pancreatitis	2 (2)	50 ± 10.0 (40–60)	5 ± 2.0 (3–7)	2:0	0	2
9.	Umbilical hernia	1 (1)	60	7	1:0	1	0
10.	Carcinoma gall bladder	1 (1)	50	14	0:1	1	0
11.	Hiatus hernia	1 (1)	64	8	1:0	1	0
12.	Gastric perforation	1 (1)	60	10	1:0	1	0

**Table 3:** Diagnosis, mean age with standard deviation (±SD), mean hospital stay standard deviation (±SD), ratio of locality of patients and management

S. no.	Diagnosis	*n* (%)	Mean age ± SD (range; y)	Mean hospital stay ± SD (range; d)	Urban–rural ratio	Management	
	Urological diseases	29 (100)				Operative	Conservative
13.	Renal calculus	17 (58.62)	34.58 ± 13.73 (18–65)	5 ± 2.72 (2–12)	12:5	15	2
14.	Ureteric calculus	11 (37.93)	36.63 ± 12.83 (24–65)	5 ± 1.04 (4–7)	7:3	8	3
15.	Pyonephrosis	1 (3.44)	28	7	0:1	1	0

**Table 4:** Diagnosis, mean age with standard deviation (±SD), mean hospital stay standard deviation (±SD), ratio of locality of patients and management

S. no.	Diagnosis	*n* (%)	Mean age ± SD (range; y)	Mean hospital stay ± SD (range; d)	Urban–rural ratio	Management	
	Breast diseases	24 (100)				Operative	Conservative
16.	Benign	19 (79.17)	34.15 ± 11.11 (18–60)	3.84 ± 1.34 (1–6)	15:4	14	5
17.	Malignant	5 (20.83)	49.20 ± 7.35 (42–63)	4.40 ± 0.80 (3–5)	3:2	5	0

**Table 5:** Diagnosis, mean age with standard deviation (±SD), mean hospital stay standard deviation (±SD), ratio of locality of patients and management

S. no.	Diagnosis	*n* (%)	Mean age ± SD (range; y)	Mean hospital stay ± SD (range; d)	Urban–rural ratio	Management	
	Perianal diseases	17 (100)				Operative	Conservative
18.	Hemorrhoids	10 (58.82)	44.5 ± 11.13 (20–55)	4.6 ± 0.8 (3–6)	2:3	7	3
19.	Fistula	4 (23.52)	35.25 ± 13.08 (25–55)	4.5 ± 1.5 (3–6)	1:1	4	0
20.	Fissure	3 (17.64)	46.3 ± 25.38 (25–82)	3.3 ± 0.47 (3–4)	2:1	3	0

**Table 6:** Diagnosis, mean age with standard deviation (±SD), mean hospital stay standard deviation (±SD), ratio of locality of patients and management

S. no.	Diagnosis	*n* (%)	Mean age ± SD (range; y)	Mean hospital stay ± SD (range; d)	Urban–rural ratio	Management	
	Thyroid diseases	10 (100)				Operative	Conservative
21.	Benign	9 (90)	42.88 ± 10.82 (27–65)	6.11 ± 2.02 (3–10)	5:4	9	0
22.	Malignant	1 (10)	36	6	0:1	1	0

**Table 7:** Diagnosis, mean age with standard deviation (±SD), mean hospital stay standard deviation (±SD), ratio of locality of patients and management

S. no.	Diagnosis	*n* (%)	Mean age ± SD (range; y)	Mean hospital stay ± SD (range; d)	Urban–rural ratio	Management	
	Others	7 (100)				Operative	Conservative
23.	Lipoma	4 (57.14)	30.5 ± 9.6 (18–45)	2.25 ± 1.08 (1–4)	3:1	4	0
24.	Sebaceous cyst	3 (42.85)	23.3 ± 6.18 (18–32)	2.33 ± 0.47 (2–3)	2:1	3	0

Of these,154 (82.35%) were managed operatively in which 131 (85.06%) patients were elective cases and 23 (14.94%) were managed in emergency and 33 (17.65%) patients treated conservatively. A total of 154 (82.35%) patients were newly admitted and 33 (17.65%) patients were follow-up patients, which include postpercutaneous nephrolithotomy for the double-J stent removal, retained stone- and stent-related complications; some patients were of wound infection postsurgery, and also some presented with seroma formation in postmastectomy. The hospital stays of patients ranged from 1 to 14 days with average stay of 6.35 days. Maximum 64.7% patients were discharged between 0- and 5-day period ( Table 8 ).

**Table 8:** Overall hospital stay of total patients in range and percentage

Hospital stay (d)	No. of patients (%)
0–5	121 (64.70)
6–10	59 (31.55)
11–15	7 (3.75)
Total	187 (100)

## Discussion

Ours is a capital city of a large state in Central India. It has a population of approximately 18 lakhs according to 2011 census. Our medical college is located in Kolar area that has population of 87,882 (45,692 males and 42,190 females). It is a 750-beded tertiary care hospital catering to a population of approximately 150,000 which include both rural as well as urban. We have well-equipped operating theaters with the latest surgical instruments including laparoscopic and endoscopic facilities.

During the study period male–female ratio of admitted patients was 4:1, despite being 1:1 in general population. This clearly shows the bias females face in our community.

It is expected that women can live longer than men, but it does not necessarily ensure a better quality of life. Many studies reported that women are sicker and more disabled that need health care support than men throughout the life cycle. It has also been suggested that women are more vulnerable, particularly where basic maternity care is unavailable. 5

The theme of International Women's Day 2020: “I am Generation Equality: Realizing Women's Rights” is aligned with the global commitment of achieving the Sustainable Development Goals related to Gender Equality by 2030.

Females tend to have poorer access to health care resources than males. These disparities are often compounded by cultural norms and expectations imposed on women. In certain societies, the females are not allowed to travel alone. This can prevent them from receiving the necessary health care. Some of the sociocultural factors that prevent women and girls to benefit from quality health services and attaining the best possible level of health include the following:

- Unequal power relationships between men and women.
- Social norms that decrease education and paid employment opportunities.
- Exclusive focus on women's reproductive roles.
- Potential or actual experience of physical, sexual, and emotional violence.

According to Weiser et al in a large community, the most common types of surgeries were one or the other type of abdominal surgeries. 6 In our study, we evaluated overall prevalence of surgical diseases in females and found that most commonly they were operated for cholelithiasis (25.13%), followed by urological disease (15.50%), breast diseases (12.83%), perianal diseases (9.09%), and thyroid diseases (5.34%).

Cholelithiasis remains one of the major causes of abdominal morbidity and mortality throughout the world. 7 Studies have shown prevalence rate of approximately 5 to 20% in Asian population. Lowest prevalence is seen in Black Africans (<5%) with gallstones being a rarity in Masi and Bantu tribes. 8 9 10 Prevalence of cholelithiasis is more in females than males. It has also been found that gallstones are more common in North India. 10 In our study, we found that incidence of cholelithiasis was 25.13% of overall patients admitted and 47% among total gastrointestinal surgery patients. Age of patients ranged from 26 to 67 years and mean age ± SD was 44.73 ± 11.59 years, which was similar to that established in literature.

Urolithiasis is the most common urological disease. Worldwide, kidney stone is the most painful and prevalent urological disorders of the urinary system. 11 In India, urolithiasis affects approximately 2 million people every year. It is a global problem spanning all geographic regions with an estimated annual incidence of 1%, prevalence of 3 to 5%, and a lifetime risk of 15 to 25%. The incidence of urolithiasis changes in different countries. In India, the “stones belt” occupies parts of Maharashtra, Gujarat, Rajasthan, Punjab, Haryana, Delhi, and States of North East. In India, 12% of the people are estimated to have urinary stones, out of which 50% may end up with loss of kidney or renal damage. Also, nearly 15% of the people of northern India are affected by urinary stones. 12 Scales et al, in a study from 1997 to 2002, found that the prevalence of stone disease had increased in female patients over the time. This has been attributed to change in lifestyle leading to obesity. 13 Lieske et al also found rising rate of prevalence of urolithiasis in female patients over a period of 30 years. 14

In our study we found that female patients admitted with urological disease were second most common group in which renal calculus 58.62% (mean age ± SD: 34.58 ± 13.73 y) was most common diagnosis followed by ureteric calculus 37.93% (mean age ± SD: 36.63 ± 12.83 y). Several authors have reported 30 to 50 years age as the period of maximum incidence of urinary calculi, which was similar to our study. 15

Third most common indication for admission was breast diseases. Out of these 79% were benign conditions like fibroadenoma. Despite this, there are very few studies on epidemiology of benign breast lumps. At least 90% of patients attending a breast clinic have benign breast diseases. 16 17 Pain is the most common symptom followed by breast lump, which brings the patients to hospital. 18 Our study reveals that there is 4:1 ratio of benign to malignant breast disease. In a study on histopathological examination of 260 cases of breast lesions, 210 (80%) were benign and 20% were malignant, which was similar to our results. 19

For decades together, cervical cancer was the most common cancer in women in India, and more deaths in women in India were attributed to cervical cancer than any other cancer. 20 However, over the past 10 years or so, breast cancer has been rising steadily, and since 2012, breast cancer became the most common cancer in women in India, ahead of cervical cancer. 21 Breast cancer seems to be more common in the younger age group in India and 52% of all women suffering from breast cancer in Mumbai are between 40 and 49 years of age. A significant number of patients are below 30 years. 22 Our patients had a mean age ± SD of 49.20 ± 7.35 years, which is in concordance with other studies.

A study done by Haas et al found that common predisposing factors for perianal disorders include constipation, pregnancy, and chronic straining. 23 24 Many patients with perianal diseases have complaints of pain, bleeding, prolapse, or itching; the most common age group is 45 to 65 years. The anorectal disorders include a wide group of pathological conditions like hemorrhoids, fissure, fistula, perianal abscess, and anal canal cancer. Goligher revealed that anal fissure is usually encountered in young and middle-aged adults, and it has no gender predilection. 25

In our study, the incidence of perianal diseases in female was seen in 17 (100%) of total cases, in which 10 (58.82%) patients had hemorrhoid, 4 (23.52%) patients had fistula, and 3 (17.64%) had fissure in ano. The age range in which patients were affected was between 20 and 82 years, and mean age was 42.01 years of overall perianal disease patients. Pregnancy being exclusive to females, we found that although females are more prone to anorectal disorders, they rarely seek surgeon's opinion due to cultural and social factors.

Thyroid diseases are arguably, among the most common endocrine disorders worldwide with female preponderance. India, too, is no exception. An estimated 42 million patients have thyroid diseases in India. Thyroid diseases, although easily diagnosed due to their location on body, require early and appropriate treatment to prevent them from becoming a burden on our health care system. A study, conducted by the Indian Council of Medical Research from 1984 to 1993 through the National Cancer Registry Program, found that there were 5,614 cases of thyroid cancer, and this included 3,617 females and 2,007 males. The six centers involved in the studies were at Mumbai, Delhi, Thiruvananthapuram, Dibrugarh, Chandigarh, and Chennai. 26

We had observed that among all admitted female patients, 10 (100%) had thyroid disease in which 9 (90%) had benign disease, whereas 1 (10%) patient had carcinoma of thyroid in 6 months of study. Thyroid cancer presented at a young age of 36 years, whereas benign disease patients had mean age ± SD of 42.88 ± 10.82 years, with a range (27–65 y). Gore et al found the mean age for benign thyroid diseases to be 36.8 ± 13.3 years, with a wide range (8–76 y), and in malignant thyroid diseases, the mean age was 42.3 years with a range of 13 to 70 years. 27

Length hospital stay (LOS) of patient is an important indicator of the use of medical services and is used to assess the efficiency of hospital management, quality of care, and functional evaluation. Decreased LOS has been associated with decreased risks of opportunistic infections and side effects of medication and with improvements in treatment outcome and lower mortality rates. Furthermore, shorter hospital stays reduce the burden of medical expenses and increase the bed turnover rate, which in turn increases the profit margin of hospitals, while lowering the overall social costs. 28 29

We found in our study that LOS ranged from 1 to 14 days, with an average of 6.35 days. Maximum patients fall in 0- to 5-day group, which had total 121 (64.70%) patients. This means approximately 65% patients were discharged within first 5 days of their admission, which indicates good hospital care and improves patient satisfaction levels.

## Conclusion

In view of the substantial lifetime prevalence of a major surgical procedure, availability of surgical care should now be a global public health concern. There is a large surgical disease burden worldwide of untreated female patients. Our findings suggest that cholelithiasis was found to be the most common surgically treated disease, followed by urological disease, breast diseases, perianal diseases, and thyroid disease in decreasing order of frequency. There is greater need for public health efforts to improve the monitoring, safety, and availability of surgical services to female patients, especially in view of their high risk for certain diseases.
